# Multimerization of Ebola GPΔmucin on protein nanoparticle vaccines has minimal effect on elicitation of neutralizing antibodies

**DOI:** 10.3389/fimmu.2022.942897

**Published:** 2022-08-24

**Authors:** Abigail E. Powell, Duo Xu, Gillie A. Roth, Kaiming Zhang, Wah Chiu, Eric A. Appel, Peter S. Kim

**Affiliations:** ^1^ Department of Biochemistry and Stanford ChEM-H, Stanford University, Stanford, CA, United States; ^2^ Department of Bioengineering, Stanford University, Stanford, CA, United States; ^3^ Chan Zuckerberg Biohub, San Francisco, CA, United States; ^4^ Division of CryoEM and Bioimaging, Stanford Synchrotron Radiation Lightsource, Stanford Linear Accelerator Center National Accelerator Laboratory, Menlo Park, CA, United States

**Keywords:** ebolavirus, glycoprotein, GPΔmucin, protein nanoparticles, subunit vaccine

## Abstract

Ebola virus (EBOV), a member of the *Filoviridae* family of viruses and a causative agent of Ebola Virus Disease (EVD), is a highly pathogenic virus that has caused over twenty outbreaks in Central and West Africa since its formal discovery in 1976. The only FDA-licensed vaccine against Ebola virus, rVSV-ZEBOV-GP (Ervebo^®^), is efficacious against infection following just one dose. However, since this vaccine contains a replicating virus, it requires ultra-low temperature storage which imparts considerable logistical challenges for distribution and access. Additional vaccine candidates could provide expanded protection to mitigate current and future outbreaks. Here, we designed and characterized two multimeric protein nanoparticle subunit vaccines displaying 8 or 20 copies of GPΔmucin, a truncated form of the EBOV surface protein GP. Single-dose immunization of mice with GPΔmucin nanoparticles revealed that neutralizing antibody levels were roughly equivalent to those observed in mice immunized with non-multimerized GPΔmucin trimers. These results suggest that some protein subunit antigens do not elicit enhanced antibody responses when displayed on multivalent scaffolds and can inform next-generation design of stable Ebola virus vaccine candidates.

## Introduction

Ebolaviruses are highly pathogenic viruses from the *Filoviridae* family that have caused over 20 outbreaks since their discovery in 1976 and they pose an ongoing threat for future human crossover events (1, 2). In the past decade, significant progress has been made in the development of prophylactic vaccines and therapeutics to treat Ebolavirus infections. However, currently authorized vaccine formulations are virus-based and to date have only been used in ring vaccination campaigns during active outbreaks (3, 4). Development of a non virus-based vaccine against Ebola could therefore expand the current tools used for fighting Ebola outbreaks and allow for prophylactic vaccine administration in areas where zoonotic crossover of filoviruses is most prevalent.

To date, two viral-based vaccines (rVSV-ZEBOV/Merck and Ad.26ZEBOV+MVA-BN-Filo/J&J) have received authorization for use in humans; both vaccines have received EMA authorization and rVSV-ZEBOV has received FDA authorization (5). rVSV-ZEBOV consists of a replicating vesicular stomatitis virus (VSV) backbone which displays the Ebola GP on the surface and requires only a single dose (6). rVSV-ZEBOV is highly protective, with efficacy values as high as 97.5% for survival in cases where symptoms of infection occurs more than 10 days post vaccination, and 88.1% regardless of when exposure occurred relative to vaccination (2, 7). Ad.26ZEBOV+MVA-BN-Filo requires administration of two doses and consists of two replication-deficient viruses, Ad.26 and modified vaccinia Ankara (MVA), which encode for a set of Ebola GPs and nucleoproteins (8, 9). This vaccine has not been tested for efficacy, but showed elicitation of robust and durable antibody levels following vaccination (9).

Despite the successful development of these vaccines, they present some key limitations related to access and distribution. Specifically, the inclusion of a replicating virus in rVSV-ZEBOV requires vaccine storage and transport at -70°C and vaccine potency decreases substantially at 4°C within two weeks after thawing (10). Since it is composed of non-replicating viruses, Ad.26ZEBOV+MVA-BN-Filo can maintain potency under refrigerated temperatures for up to 12 months (11), however the two-dose regimen of Ad.26ZEBOV+MVA-BN-Filo presents critical logistical challenges for administration as repeated access to patients in resource limited settings is often difficult. Due to these reasons, neither vaccine is currently administered as a means of outbreak prevention, but have only been used in ring vaccination strategies to minimize and protect against active outbreaks (4). Development of a more shelf-stable vaccine could therefore increase prophylactic administration and provide an additional tool in the fight against Ebola.

While virus-based vaccines typically elicit more robust and durable immune responses as compared to protein subunit vaccines, subunit vaccines often afford key stability and storage benefits for a vaccine formulation (12). Additionally, immune responses to subunit vaccines can be enhanced with the addition of adjuvants and/or engineering strategies that facilitate multivalent presentation of the antigen. Prior efforts to develop a nanoparticle vaccine against Ebola demonstrated that multivalent presentation of GP on both particles extracted from insect cells as well as polymer-based particles elicited protective antibody responses in rodents (13, 14). Recent work from He et al. has also demonstrated the ability to elicit Ebola neutralizing antibodies in mice and rabbits using protein-based nanoparticle scaffolds functionalized with engineered GP trimers (15).

Most vaccine efforts for development of Ebola vaccine candidates have focused on use of the sole surface protein found on the virus, GP, to elicit a protective antibody response. GP is expressed as a single polypeptide which is then cleaved by furin to form GP1/GP2 subunits, yielding a trimer of heterodimers (16–18). The receptor binding domain is contained within GP1 whereas GP2 contains the fusion machinery necessary for fusion with the endosomal membrane following binding to its receptor, Niemann-Pick C1 (19–22). Prior to host cell entry, GP contains two heavily glycosylated domains, the mucin-like domain (MLD) and the glycan cap, which are proteolytically removed by host-cell cathepsins (23, 24). Antibodies targeting the MLD rarely exhibit viral neutralization activity (25), presumably because this domain is shed prior to receptor binding, and because the heavily glycosylated MLD dramatically reduces protein expression levels (26). Due to this, we designed our multivalent GP-functionalized nanoparticle vaccine antigens using a truncated form of GP, GPΔmucin, which lacks the MLD (18, 27).

**Figure 1:**
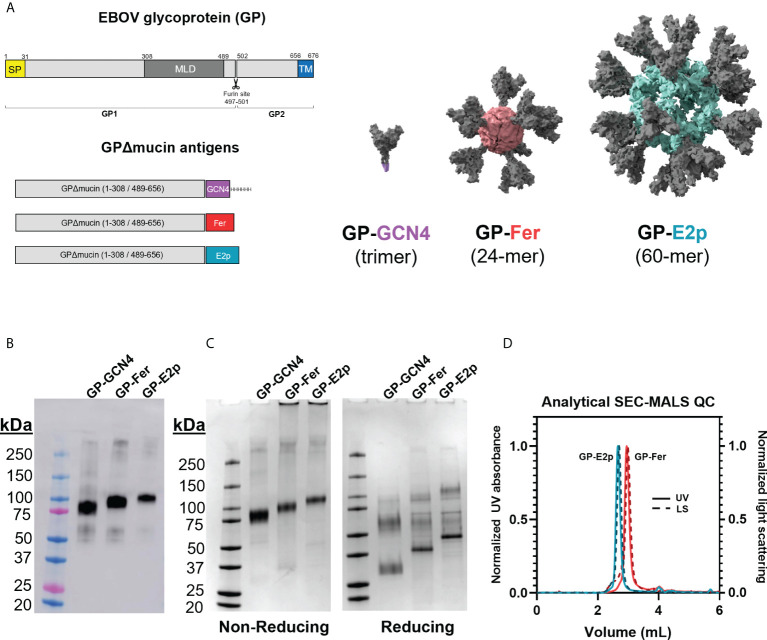
Protein nanoparticles functionalized with GPΔmucin can be expressed in mammalian cells and purified to homogeneity. **(A)** GPΔmucin antigens were designed using the Ebola virus (Mayinga, Zaire, 1976) sequence. The domains of GP are shown in the top schematic and indicate the relative positions of the signal peptide (SP), the mucin-like domain (MLD), and the transmembrane domain (TM). GPΔmucin soluble antigens were designed by removing the sequence encoding amino acids 309-488. The TM domain was also removed and replaced with either a GCN4 trimerization domain (GP-GCN4), a ferritin subunit (GP-Fer), or an E2p subunit (GP-E2p). For the GP-Fer and GP-E2p constructs, an SGG linker was inserted in between GPΔmucin and the nanoparticle subunit. **(B)** Expi supernatants from cells expressing GP-GCN4, GP-Fer, and GP-E2p were blotted for the presence of antigen using mAb114, a GP1-reactive monoclonal antibody. Western blots indicated that expression levels were not perturbed by nanoparticle functionalization of GPΔmucin. **(C)** GPΔmucin were assessed for purity using SDS-PAGE analysis under non-reducing (left) and reducing (right) conditions and stained with GelCode Blue stain. Non-reducing conditions yield a single band whereas reducing conditions show multiple species, indicating that GPΔmucin antigens are cleaved at the native furin site between GP1 and GP2. **(D)** SEC-MALS analysis of GP-E2p and GP-Fer following purification indicate that the nanoparticles are homogenous and do not exhibit aggregation.

For many antigens, multimerization leads to dramatically enhanced neutralizing antibody responses following vaccination. As a potential strategy for a protein-based subunit vaccine against Ebola, we present here the design of self-assembling protein nanoparticles which can be readily produced in mammalian cells and purified to homogeneity. We separately fused GPΔmucin to two self-assembling protein nanoparticles, the 24-mer *H. pylori* ferritin subunit (GP-Fer) (28) and the 60-mer *B. stearothermophilus* 2-oxo acid dehydrogenase subunit E2 (GP-E2p) (29), yielding nanoparticles displaying 8 or 20 GPΔmucin trimers (**Figure 1A**). To understand the impacts of GP multimerization on antibody responses in the context of vaccination, we also included a GPΔmucin trimer alone control in our studies in which we replaced the nanoparticle subunits with a GCN4 trimerization domain (GP-GCN4). We demonstrate that GPΔmucin can be stably displayed on nanoparticles and show using biolayer interferometry (BLI) that relevant neutralizing epitopes remain intact. Immunization of mice with the GPΔmucin nanoparticles revealed that while the multivalent vaccines elicited a more rapid antigen-specific antibody response compared to trimer alone, under these specific conditions, increased valency had minimal effect on neutralizing antibodies levels. While multivalent antigen presentation is often associated with enhanced levels of antibody elicitation following vaccination (28, 30–32), these results indicate that this effect is minimal in the context of GPΔmucin. The results presented here therefore provide important considerations for development of current and future nanoparticle-based vaccine formulations.

## Materials and methods

### Molecular cloning and plasmid production

DNA encoding the GP ectodomain (residues 1-656) from EBOV (Mayinga, Zaire, 1976) with the mucin-like domain removed (residues 309-489) and the transmembrane domain replaced with a GCN4 trimerization domain, an AviTag, and a hexahistidine tag [amino acid sequence from (27)] was ordered as a gene block fragment from Integrated DNA Technologies (IDT). Gene blocks encoding the *H. pylori* ferritin (residues 5-168) subunit and the E2p subunit (residues 1-242) were also ordered from IDT. GP-Fer and GP-E2p were constructed by PCR amplifying GPΔmucin and each nanoparticle subunit fragment off of the gene blocks followed by a stitching PCR. Both nanoparticle constructs contained a Ser-Gly-Gly linker between GPΔmucin and the nanoparticle subunit. The GP-GCN4, GP-Fer, and GP-E2p fragments were then inserted into a mammalian protein expression vector (pADD2) using In-Fusion (Takara).

For production of GP-pseudotyped lentiviral particles, a gene block encoding full-length EBOV GP (Mayinga, Zaire, 1976) with transmembrane domain included was obtained from IDT (EBOV). EBOV GPΔmucin containing the transmembrane domain was constructed by PCR amplifying the ectodomain from the GP-GCN4 parent construct and the transmembrane domain from the full-length EBOV GP construct and then fusing these fragments using a stitching PCR step. The full-length EBOV GP and EBOV GPΔmucin fragments were inserted into a linearized pcDNA3.1 plasmid for use in lentivirus production. The helper plasmids used in virus production, pHAGE-CMV-Luc-IRES, HDM-Tat1b, HDM-Hgpm2, and pCMV-Rev1b were obtained as a kind gift from Dr. Jesse Bloom (Fred Hutch).

DNA encoding antibody V_H_ and V_L_ domains was ordered as gene block fragments from IDT (mAb114) (27, 33) or Twist (ADI-15742 and ADI-16061) (34), PCR amplified, and inserted into plasmids containing VRC01 heavy chain and light chain constant domains using InFusion.

All plasmids were sequence confirmed using Sanger sequencing prior to use. Plasmids were propagated using Stellar cells (Takara) grown in 2x YT media with carbenicillin, with the exception of the mAb plasmids which were grown in kanamycin. Prior to mammalian cell transfection, plasmids were isolated from bacteria using maxi-prep or midi-prep columns (Machery Nagel) and sterilized using a 0.22-µm syringe filter in a biosafety cabinet.

### Protein expression and western blotting from cell supernatants

All proteins were expressed and purified from Expi293F suspension cells. Expi293F cells were cultured in a 66%/33% mixture of Freestyle/Expi medium (ThermoFisher) in polycarbonate shaking flasks under continuous shaking at 120 rpm in a humidified 8% CO_2_ incubator. Transfection complexes were formed by adding 15 µg DNA to 3 mL culture media followed by addition of 39 µL FectoPro transfection reagent (Polyplus) per 30 mL cells transfected. Antibodies were produced by transfecting a 1:1 ratio of HC and LC plasmids. Complexes were incubated at room temperature for 10 min and then added to cells at 3 – 4 x 10^6^ per mL density. Cells were boosted immediately following transfection with 0.4 g/L D-glucose and 3 mM valproic acid, final concentrations.

To monitor protein expression levels directly from culture supernatant, small-scale expressions (10 mL Expi293F cells) were transfected as described. Three days post transfection, cells were harvested *via* centrifugation at 7000 *xg* for 15 min and supernatants were filtered through a 0.22-µm filter. Supernatants were diluted with Laemmli loading buffer, boiled at 95°C, and then loaded (1 µL supernatant final) on a 4-20% Mini-PROTEAN TGX gel. Proteins were transferred onto a nitrocellulose membrane and blots were blocked in PBS [pH 7.4] with 0.1% Tween-20 (PBST) and 5% milk added. After blocking blots were washed with PBST and then incubated with in-house made mAb114 primary antibody diluted to ~2 nM in PBST. Blots were washed with PBST and then incubated with rabbit anti-human IgG HC & LC HRP secondary antibody, diluted 1:10,000 in PBST. Blots were developed using Pierce ECL western reagent and imaged using a GE Amersham Imager 600 in chemiluminescence mode.

### Protein purification

Cells were harvested 3-6 days post transfection by centrifugation at 7000 *xg* for 15 min and culture supernatants were filtered through a 0.22-µm filter prior to purification. GP-Fer and GP-E2p culture supernatants were concentrated using an AKTA Flux S tangential flow filtration system with a 10 kDa molecular weight cutoff (MWCO) hollow fiber cartridge (UFP-10-E-4MA) and then buffer exchanged into 20 mM Tris [pH 8.0] *via* dialysis using regenerated cellulose 100 kDa MWCO tubing (Spectrum Labs). Dialyzed supernatants were then loaded onto a HiTrapQ anion exchange column (GE) and eluted with a sodium chloride gradient. Nanoparticle-containing fractions were identified by western blots probed with mAb114, pooled, and then subsequently purified *via* size-exclusion chromatography on a SRT SEC-1000 column equilibrated in PBS [pH 7.4].

GP-GCN4 trimers were purified by batch incubating cell supernatant with HisPur NiNTA resin (ThermoFisher) at 4°C. Resin/supernatant mixtures were then added to chromatography columns with frit barriers and resin was washed with > 10 column volumes of wash buffer (10 mM imidazole/1X PBS [pH 7.4]). Protein was eluted with 250 mM imidazole/1X PBS [pH 7.4], concentrated using 50 kDa MWCO Amicon spin concentrators, and purified *via* size-exclusion chromatography using a GE Superose 6 Increase 10/300 GL column in 1X PBS [pH 7.4]. Following SEC, antigen-containing fractions were identified using SDS-PAGE analysis on 4-20% Mini-PROTEAN TGX gels followed by GelCode blue staining (ThermoFisher). For immunization samples, purified fractions were pooled, supplemented with 10% glucose (final concentration), filtered with a 0.22-µm syringe filter in a biosafety cabinet, snap frozen, and stored at -80°C until use.

### Analytical SEC-MALS and DSF

SEC-MALS analysis of GP-Fer and GP-E2p particles was performed using an Agilent 1260 Infinity II HPLC (Agilent) coupled to a miniDAWN and Optilab detectors (Wyatt Technologies) for light scattering and refractive index analysis. Approximately 10 µg purified nanoparticles were loaded onto an SRT SEC-1000 4.6 x 300 mm column in 1X PBS [pH 7.4] at a flow rate of 0.35 mL/min. ASTRA 7.3.2 software (Wyatt Technologies) was used to calculate molecular weights using the light scattering and refractive index data for each GP nanoparticle.

Differential scanning fluorimetry was performed using a Prometheus NT.48 (NanoTemper). Proteins were loaded into capillary tubes and were subjected to a temperature gradient from 20°C to 90°C at a rate of 1°C per min. Melt curves were obtained by plotting the ratio of fluorescence at 350/330 nm as a function of temperature.

### Cryo-EM data acquisition and single-particle 2D classification

GP-Fer and GP-E2p were diluted to ~1 mg/mL (protomer concentration) in 1X PBS [pH 7.4]. Three µL of sample was applied to glow-discharged 200-mesh R2/1 Quantifoil copper grids coated with thin carbon film. The grids were blotted for 1 s and rapidly cryocooled in liquid ethane using a Vitrobot Mark IV (Thermo Fisher Scientific) at 4°C and 100% humidity. The grid was imaged in a Titan Krios cryo-electron microscope (Thermo Fisher Scientific) operated at 300 kV at a magnification of 75,000× (corresponding to a calibrated sampling of 1 Å per pixel) for both samples. Micrographs were recorded by EPU software (Thermo Fisher Scientific) with a Falcon 4 direct electron detector, where each image was composed of 40 individual frames with an exposure time of 8.5 s and an exposure rate of 5.85 electrons per second per Å^2^. A total of 337 movie stacks for GP-Fer and 332 movie stacks for GP-E2P were collected. All movie stacks were first imported into Relion for image processing. The motion-correction was performed using MotionCor2 and the contrast transfer function (CTF) was determined using CTFFIND4. All particles were autopicked using the NeuralNet option in EMAN2. Then, particle coordinates were imported to Relion, where the 2D class averages were performed.

### BLI

BLI experiments were performed using an OctetRed 96 (ForteBio). GP-Fer, GP-E2p, and GP-GCN4 were diluted to 100 nM (protomer concentration) and mAbs were diluted to 200 nM in Octet buffer (0.5% bovine serum albumin, 0.02% Tween-20, 1X Dulbecco’s phosphate-buffered saline [pH 7.4]). Antigens and mAbs were plated in 96-well black-bottom plates. Anti-human Fc coated sensor tips were pre-equilibrated in Octet buffer prior to binding experiments. For each binding experiment, sensor tips were dipped into mAb-containing wells and subsequently dipped into antigen-containing wells. Data was background subtracted using signal from a tip dipped into a well containing buffer only. Curves were fit in GraphPad Prism 9.1.0 with a kinetic association / dissociation equation to obtain comparative estimates for k_on_ values for each GP antigen.

### Mouse immunizations

Female C57BL/6 mice (8 weeks old) were obtained from Charles River and maintained at Stanford University according to the Public Health Service Policy for “Human Care and Use of Laboratory Animals” following a protocol approved by the Stanford University Administrative Panel on Laboratory Animal Care (APLAC-32109). Mice were immunized at day 0 with 5 µg GP antigen (GP-Fer, GP-E2p, or GP-GCN4, *n* = 5 per group) adjuvanted with 10 µg monophosphoryl lipid A (Invivogen) and 10 µg Quil-A (In vivogen) *via* subcutaneous injection (100 µL per injection). Adjuvants were mixed with antigen immediately prior to injections. Mice were immunized with a second dose 18 weeks post primary immunization with 5 µg GP-GCN4 in DPBS as a proxy for viral challenge. At interim timepoints, blood was collected from the tail vein (collection time points shown in **Figure 3A**). Final bleeds were collected *via* cardiac puncture. Interim and final blood samples were processed using clotting activator serum collection tubes (Sarstedt) according to manufacturer’s recommendations. Serum was aliquoted and stored at -80°C until use.

### ELISAs

Mouse serum ELISAs were performed using MaxiSorb 96-well plates (ThermoFisher). Antigens (either GPΔmucin or FL-GP tagged with a foldon domain) were diluted to 1.5 µg/mL in 1X PBS [pH 7.4] and incubated on plates overnight at 4°C (50 µL volume per well). Plates were then washed 3X with 300 µL PBST using a BioTek plate washer. After antigen coating, plates were blocked by adding 200 µL ChonBlock Blocking/Diluent ELISA buffer (Chondrex) and incubating overnight at 4°C. ChonBlock was removed manually and plates were then washed 3X with 300 µL PBST. Mouse serum was diluted in PBST with a 6-point dilution series from 1:100 to 1:10,000,000 and added to blocked plates and incubated at room temperature for 1 hr. Following serum incubation, plates were washed 3X with 300 µL PBST and were subsequently incubated with goat anti-mouse IgG HRP secondary antibody (BioLegend 405306) diluted 1:10,000 in PBST for 1 hr at room temperature. Plates were then washed 6X with 300 µL PBST and developed by adding 50 µL 1-Step Turbo TMB-ELISA substrate (ThermoFisher) for 6 minutes and then quenched with 50 µL 2M sulfuric acid.

ELISAs were performed in experimental duplicate for each mouse sample at each time point. Data was analyzed using GraphPad Prism 9.1.0 by fitting each dilution series with a three-parameter dose-response curve. EC_50_ values for each mouse at each time point was then plotted for comparison and statistical analysis was performed in GraphPad Prism 9.1.0 using ordinary one-way ANOVA with Tukey’s multiple comparisons test.

### GP-pseudotyped lentivirus production and viral neutralization

Lentivirus particles pseudotyped with full-length and mucin-deleted Ebola GP were produced as previously described for SARS-CoV-2 pseudotyped viruses using calcium phosphate transfection (31, 35). Briefly, 6 million HEK293T cells cultured in D10 media (DMEM + additives: 10% fetal bovine serum, L-glutamate, penicillin, streptomycin, and 10 mM HEPES) were seeded in 10 cm dishes one day prior to viral transfection. Viral transfection plasmids consisting of a pHAGE-Luciferase packaging vector (10 µg), lentiviral helper plasmids (HDM-Hgpm2, HDM-Tat1b, and pRC-CMV_Rev1b; 2.2 µg each), and Ebola GP expression plasmid (3.4 µg) were added to 0.5 mL H_2_O and then diluted with 2X HEPES-buffered saline [pH 7.0] (Alfa Aesar) to a final volume of 1 mL. 100 µL 2M calcium chloride was then added dropwise to diluted DNA and transfection complexes were incubated for 20 min at room temperature and then added dropwise to cells. Media was aspirated off cells ~18 – 24 hr post transfection and fresh media was added. Viral supernatants were harvested 72 hrs post transfection by centrifugation at 300 xg for 5 min followed by filtering through a 0.45-µm syringe filter. Lentivirus stocks were aliquoted and stored at -80°C until use.

For serum neutralization assays, HEK293T cells were used as targets of infection. One day prior to infections, HEK293T cells were plated at 10K cells per well in D10 media in white-walled, clear bottom tissue culture-treated plates. Prior to neutralization assays, mouse serum was heat inactivated for 30 min at 56°C and then diluted in D10 media. For single-point neutralization assays, serum was diluted to 1:300 final concentration and for serum dose-responses curves, serum was assessed with a 6-point dilution series from 1:50 to 1:156,250. GP-pseudotyped lentiviruses were diluted with D10 media, supplemented with Polybrene, and added to diluted serum samples (5 µg/mL final Polybrene). Virus/serum samples were incubated at 37°C for 1 hr. Media was then aspirated off of HEK293T cells and replaced with virus/serum dilutions. Cells were incubated with virus/serum samples for 72 hrs and then lysed and assessed for luciferase activity using BriteLite (PerkinElmer). Luminescence signal was recorded using a BioTek plate reader.

For analysis of neutralization results, luminescence values were normalized using the average values for cells only (0% infectivity) or virus only (100% infectivity) in GraphPad Prism 9.1.0. Single-point analyses were then plotted as % neutralization as a function of time post immunization. Dilution series were analyzed by fitting normalized data with a three-parameter dose-inhibition curve. NT_50_ values obtained for each mouse were then plotted for comparison and statistical analysis was performed in GraphPad Prism 9.1.0 using ordinary one-way ANOVA with Tukey’s multiple comparisons test.

## Results

### GPΔmucin nanoparticles can be expressed in mammalian cells and purified to homogeneity

We designed GP-functionalized nanoparticles by fusing GP (Mayinga, Zaire, 1976) residues 1-308 and 489-656 (with a covalent linkage directly between amino acids 308 and 489) (27) to the N-terminus of either ferritin or E2p separated by an SGG linker (**Figure 1A**). In addition to the ferritin and E2p nanoparticles, we also generated a trimeric GPΔmucin form by substituting a GCN4 trimerization domain (27) in place of the nanoparticle subunit. Our GP construct removes the mucin-like domain (residues 309-488) but retains the native furin cleavage site (residues 497-501). GP is expressed as a single polypeptide which is then cleaved by furin into GP1 and GP2 subunits which are covalently bound *via* a disulfide bond between cysteines 53 and 609 (16–18).

GPΔmucin contains 9 N-linked glycosylation sites (36) and thus production in mammalian cells is important for generating glycosylated antigens. We expressed GP-GCN4, GP-Fer, and GP-E2p in Expi293F suspension mammalian cells and monitored protein expression levels using a western blot blotted with the GP-specific monoclonal antibody mAb114 (**Figure 1B**) (27, 33). Importantly, we observe that the nanoparticle functionalization does not impact expression levels compared to the expression levels of trimer alone.

We obtained homogenous nanoparticle samples following purification of the GP nanoparticles using anion exchange and size-exclusion chromatography as determined by SDS-PAGE and SEC-MALS analysis (**Figures 1C, D**). Given that the native furin cleavage site is left intact in our GPΔmucin construct, we observe cleavage and separate migration of the GP1/GP2 subunits under reducing SDS-PAGE conditions. Interestingly, the nanoparticle samples, GP-Fer and GP-E2p, exhibit less efficient cleavage as compared to GP-GCN4, with these samples containing a greater fraction of uncleaved GP under reducing SDS-PAGE conditions (**Figure 1C**). Despite the incomplete cleavage of GP seen with the GP-functionalized nanoparticles, size-exclusion chromatography multi-angle light scattering (SEC-MALS) of GP-Fer and GP-E2p reveal highly homogenous nanoparticle samples following purification (**Figure 1D**) and thus we do not expect the level of furin cleavage to impact the presentation of GPΔmucin on nanoparticles. Using the refractive index and light scattering signal from the SEC-MALS analysis, we experimentally determined molecular weight values of 2.1 MDa and 5.9 Mda for the GP-Fer and GP-E2p nanoparticles (**Supplementary Figure S1**). These values are comparable to the theoretical molecular weights based on amino acid sequence (1.7 and 4.6 MDa) and the experimentally determined values are likely large due to the presence of N-linked glycans on the GPΔmucin moiety.

### GPΔmucin nanoparticles are thermally stable, contain homogenous particle core structures, and exhibit efficient binding to conformation specific antibodies

**Figure 2:**
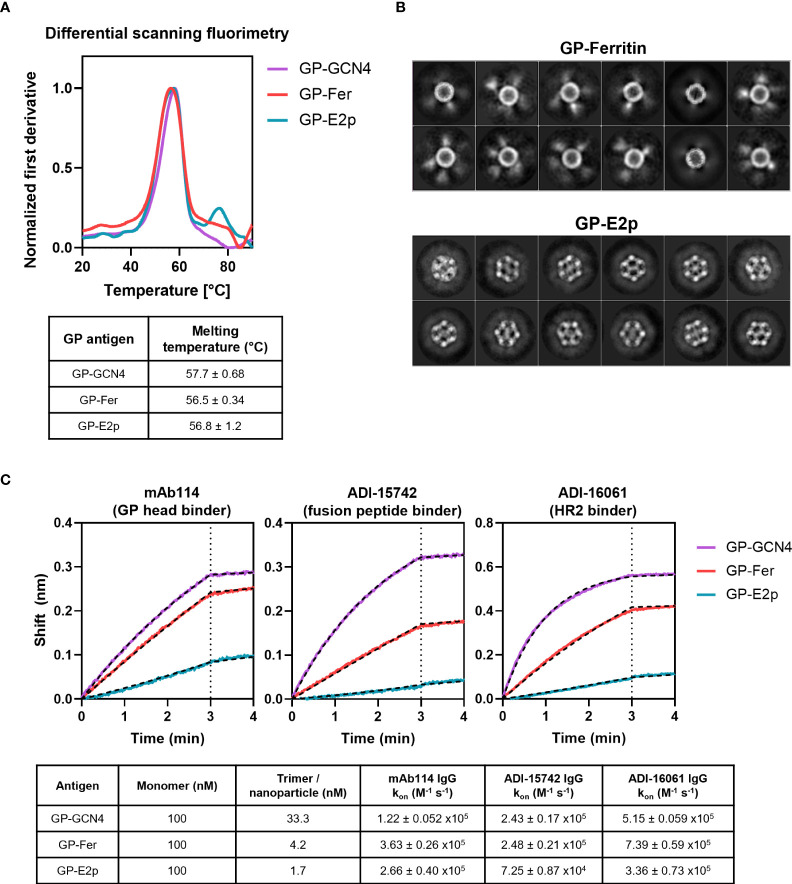
Stability and antigenicity of GPΔmucin are not perturbed when displayed on protein nanoparticles. **(A)** Stability of GP-GCN4, GP-Fer, and GP-E2p were assessed using differential scanning fluorimetry using a melting gradient from 20°C to 90°C at a rate of 1°C per minute. Melting temperatures were obtained from the Prometheus software using the fit of the first derivative of the 350/330 nm fluorescence ratio as a function of temperature. **(B)** 2D class averages from cryo-EM analysis of GP-Fer and GP-E2p. **(C)** Binding of mAbs to GP-GCN4, GP-Fer, and GP-E2p was assessed using purified mAbs immobilized on anti-human Fc biosensor tips. mAbs were dipped into antigen (100 nM, subunit concentration) and association was monitored for 3 min. Binding experiments were performed in experimental duplicate and k_on_ values were calculated by fitting curves with an association/dissociation kinetic equation in GraphPad Prism. Binding curves from one representative replicate are shown with curve fits plotted as hashed black lines. Average k_on_ values for each binding reaction with standard deviation are shown in table.

To confirm that the fusion of GPΔmucin to both ferritin and E2p did not disrupt the stability or structure of the trimer, we used differential scanning fluorimetry (DSF) to determine the melting temperatures (T_m_) of each antigen. As seen in **Figure 2A**, DSF revealed a T_m_ of ~57°C for GP-GCN4, GP-Fer, and GP-E2p with less than 1.5°C difference between each antigen, suggesting that the display on nanoparticles did not destabilize or perturb GPΔmucin.

In addition to assessing the stability of GPΔmucin, we visualized the ferritin and E2p functionalized particles using cryo-electron microscopy (cryo-EM) to confirm particle homogeneity. The 2D class averages from our cryo-EM data sets demonstrated that we could observe the core particles for both the ferritin and E2p nanoparticles (**Figure 2B**). The GP-Fer particles exhibit smeared density surrounding the core particle which corresponds to the GPΔmucin on the surface of the particle. Density can also be observed surrounding the GP-E2p core particle, however it is less distinguishable likely due to the increased number of GPΔmucin trimers on the surface as compared to ferritin.

Importantly, we wanted to demonstrate that critical neutralizing epitopes on GPΔmucin remained in relevant conformations when displayed on ferritin and E2p nanoparticles. We expressed and purified three conformation specific neutralizing antibodies that recognize different epitopes on GPΔmucin. Specifically, we chose mAb114 (27, 33) which recognizes an epitope in the GP1 head region, ADI-15742 (37) which binds to the fusion peptide, and ADI-16061 (37) which binds to the HR2 domain of GP2. We immobilized the antibodies on anti-human-Fc sensor tips and then dipped them into wells containing either GP-GCN4, GP-Fer, or GP-E2p. It is important to note that in this assay, the antigens were added at an equivalent molar monomer concentration of 100 nM. Due to the varied valency of each antigen, this yields a final trimer concentration of 33.3 nM GP-GCN4, and final nanoparticle concentrations of 4.2 nM GP-Fer and 1.7 nM GP-E2p. When we fit each binding curve with a kinetic association / dissociation equation, we observe similar k_on_ values (**Figure 2C**) for each of the three antigens, indicating that the mAbs can comparably recognize and bind their epitopes whether or not GPΔmucin is displayed on a nanoparticle surface.

### Immunization with GPΔmucin nanoparticles leads to rapid elicitation of antigen-specific antibodies

**Figure 3:**
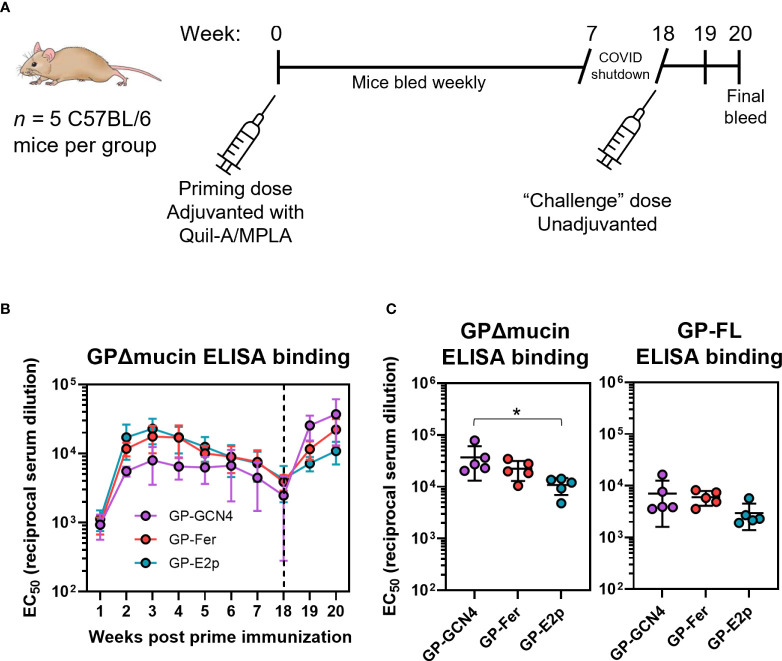
GPΔmucin nanoparticles rapidly elicit antigen-specific antibodies *in vivo.* **(A)** Immune responses to GPΔmucin antigens were assessed in C57BL/6 mice *(n =* 5 per antigen). Mice were immunized at day 0 with 5 µg antigen formulated with 10 µg Quil-A and 10 µg MPLA. Blood was collected weekly from weeks 1 to 7 post prime immunization. Mice were challenged with an unadjuvanted dose of 5 µg GP-GCN4 as a proxy for pathogen exposure. Mice were bled one week post second dose and sacrificed two weeks post second dose. **(B)** Weekly bleeds were analyzed using ELISAs to compare antigen-specific responses between GP-GCN4, GP-Fer, and GP-E2p. Each point represents the mean EC_50_ titer for each group at each time point and error bars represent the standard deviation. ELISAs were performed in experimental duplicate. **(C)** Final bleeds were assessed for both GPΔmucin and FL GP titers *via* ELISA. Each point represents the EC_50_ value per animal and error bars represent the standard deviation. Statistical analysis was performed using ordinary one-way ANOVA with Tukey’s multiple comparisons test (* = *p* ≤ 0.05).

To characterize the *in vivo* response to the GPΔmucin nanoparticles, we immunized mice (*n* = 5) with 5 µg of either GP-Fer, GP-E2p, or GP-GCN4 formulated with 10 µg monophosphoryl lipid A (MPLA) and 10 µg Quil-A adjuvants. This adjuvant combination has been shown to elicit robust and durable immune responses in the context of immunization (38–40) and we have previously demonstrated that Quil-A+MPLA facilitated elicitation of strong neutralizing antibody responses in the context of nanoparticle vaccines against SARS-CoV-2 (31). We collected serum weekly from weeks 1 to 7 post vaccination, and again at 18 weeks post vaccination (**Figure 3A**). We performed enzyme-linked immunosorbent assays (ELISAs) to compare the levels of GPΔmucin antibodies elicited by each antigen (**Figure 3B**). We performed these experiments using GPΔmucin containing a foldon trimerization domain to avoid detecting antibodies raised against either the GCN4 on the GPΔmucin trimer or the ferritin or E2p nanoparticle subunits. In response to immunization with just a single dose of antigen, we observed rapid elicitation of antigen-specific antibodies in all three antigen groups. The antibody levels for all groups peaked around week 3 post immunization and declined to similar levels across all three groups by week 18 post immunization (**Figure 3B**).

As a proxy for pathogen exposure, we administered 5 µg GP-GCN4 in the absence of adjuvant to all animals following serum collection of the week 18 timepoint to determine if we could observe a memory recall response. The goal of this injection was not to act as a booster dose, but instead to observe the antibody response in mice as a result of exposure to the GPΔmucin antigen without the influence of immunomodulatory adjuvants present. Since we could not challenge with live-virus, we chose to immunize with trimer antigen alone in all groups to test if the recall response would be enhanced in animals that had initially been administered multimerized antigens. While this approach cannot estimate survival following pathogen exposure as a challenge study would, it could help to determine whether or not the initial dose of adjuvanted antigen was sufficient to generate a memory response upon later exposure to Ebola antigen. We observed an increase in antibody levels in all groups which was the highest following antigen exposure in the GP-GCN4 group. When we quantified the EC_50_ values for antigen-specific antibody binding two weeks following the non-adjuvanted dose, we surprisingly observed an inverse trend between antigen multimerization and GPΔmucin-specific antibody levels (**Figure 3C**). Additionally, this trend was also consistent when we tested antibody levels against the full-length GP antigen containing the mucin-like domain.

### GPΔmucin nanoparticles elicit similar levels of neutralizing antibodies compared to trimers alone

**Figure 4:**
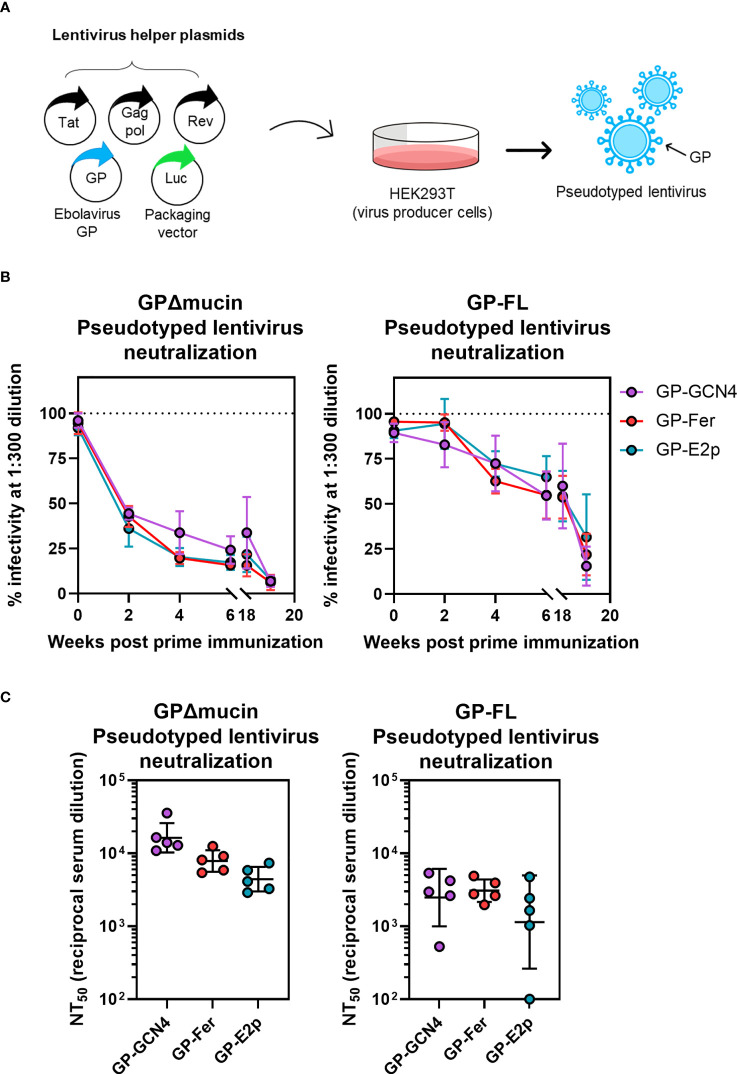
Sera from mice immunized with GPΔmucin antigens neutralize GP-pseudotyped lentiviruses. **(A)** Lentiviruses pseudotyped with GPΔmucin were produced by transfecting a lentiviral packaging vector as well as plasmids expressing lentiviral helper proteins and GPs into HEK239T cells. Viruses were harvested from HEK293T supernatant and used to assess viral entry inhibition *via* expression of a luciferase reporter gene. **(B)** Viral neuralization of both GPΔmucin and FL GP pseudotyped viruses was assessed at a single dilution point (1:300) with serum collected at weeks 2, 4, 6, 18, and 20 post prime immunization. Each point represents the mean % infectivity in the presence of 1:300 serum dilution for each group at each time point and error bars represent the standard deviation. **(C)** Final bleeds were assessed for neuralization of both GPΔmucin and FL GP pseudotyped viruses using a 6-point dilution series. Dilution curves were fit to obtain NT_50_ values; each point represents the average NT_50_ for a single mouse, the line represents the geometric mean per group, and error bars represent the geometric standard deviation. Statistical analysis was performed using ordinary one-way ANOVA with Tukey’s multiple comparisons test and no statistically significant differences were found between the three antigen groups.

In addition to quantifying antibody levels induced by immunization *via* ELISA, we also sought to determine how elicitation of virus neutralizing antibodies differed among antigens with different levels of multimerization. Ebola is a BSL-4 pathogen and thus considerable resources are required to perform live-virus neutralization experiments. Previous work has demonstrated that non-pathogenic, pseudotyped viruses displaying the Ebola GP protein can be used to quantify viral neutralization and these systems have shown good agreement with corresponding live-virus experiments (41). Thus, we created lentivirus particles pseudotyped with either the full-length Ebola GP or with GPΔmucin to compare levels of viral neutralizing antibodies elicited by our designed antigens (**Figure 4A**). We validated our pseudovirus neutralization assay using three previously described mAbs [mAb114 (27, 33), ADI-15742 (37), and ADI-16061 (37)] to demonstrate that IC_50_ values obtained in our neutralization system were consistent with published values (**Supplementary Figure S2**).

For temporal serum samples collected between weeks 1 and 18 post-initial immunization, we assessed neutralizing capacity at a single serum dilution (1:300) to compare neutralization levels across all three antigen groups (**Figure 4B**). Surprisingly, we observed a striking similarity in the level of neutralization elicited by all three antigens between weeks 1 and 18 post-initial immunization (**Figure 4B**) despite a ~0.5-log higher level of antigen-specific antibodies observed in the GP-Fer and GP-E2p groups at these time points compared to GP-GCN4 (**Figure 3B**). The similar level of neutralization potency across groups was present for neutralization of viruses pseudotyped with both GPΔmucin (**Figure 4B**, left) and full-length GP (**Figure 4B**, right), however neutralizing antibody levels against GPΔmucin appeared to be generated more quickly following a single dose of antigen. Virus neutralization with a 1:300 dilution of serum reached 50% for GPΔmucin-pseudotyped viruses by week 2 post immunization, whereas the same level of neutralization of full-length GP-pseudotyped virus was not reached until 6 weeks post immunization. At week 18, we observed approximately 50% neutralization of full-length GP pseudotyped lentivirus from all three antigen groups (**Figure 4B**, right), indicating that these GPΔmucin antigens elicit a 50% neutralization titer (NT_50_) value of 1:300 serum dilution with just a single dose and are durable to at least 4 months post immunization.

For the final immunization time point, we generated viral neutralization curves using serum from each mouse to calculate an NT_50_ value for neutralization of both FL GP- and GPΔmucin-pseudotyped lentivirus infection. Consistent with the ELISA results, we observed a decreasing trend between serum neutralization levels and antigen multimerization level (**Figure 4C**, left). Interestingly, serum neutralization of FL GP-pseudotyped lentivirus was nearly equivalent across all three groups. Additionally, NT_50_ values for neutralization of full-length GP-pseudotyped virus were ~0.5-log lower than those required to neutralize GPΔmucin-pseudotyped virus. Although the sample sizes are too small to be definitive, GP-Fer may elicit a more consistent neutralizing response with full-length pseudotyped virus.

## Discussion

Ebola virus disease is associated with severe infections with high mortality and in recent years Ebola outbreaks have increased in prevalence and scale. Substantial progress has been made in development of therapeutics and vaccines for Ebola, and two virus-based vaccines have been approved for human use. However, these vaccines require cold-chain storage and transport and/or multiple doses for efficacy thus their use has been limited to ring vaccination in the context of active outbreaks. Therefore, expanding the repertoire of vaccine candidates that protect against Ebola, specifically focused on non-viral formulations, could allow for prophylactic vaccine administration in areas of high prevalence for viral emergence as a means for preventing future outbreaks. We investigated the use of protein nanoparticle scaffolds to design multivalent antigens displaying Ebola GPΔmucin. GPΔmucin displayed on protein nanoparticles with valency of either 8 (ferritin) or 20 (E2p) trimers per particle showed equivalent expression levels, stability, and antigenicity to GPΔmucin trimer alone. Interestingly, though the GPΔmucin nanoparticles exhibited a more rapid antigen-specific antibody response in mice, neutralizing antibody levels of both multimeric and trimeric GPΔmucin antigens were not significantly different. Therefore, while protein antigens based on Ebola GP can elicit neutralizing antibodies with a single dose, multimerization may not be required for an efficacious subunit Ebola vaccine.

Despite discovery of the virus nearly five decades ago, efforts toward development of an Ebola vaccine did not garner widespread global attention until the unprecedented outbreak that occurred in West Africa from 2013 to 2016. Spurned by this outbreak, rapid development of the first clinically approved vaccine against Ebola, rVSV-ZEBOV, was a momentous achievement and has set the standard for Ebola vaccine candidate development moving forward. However, as evidenced by the ongoing COVID-19 pandemic, management and prevention of pandemic pathogens requires a diverse set of tools including an array of vaccine and therapeutic strategies. Unforeseen circumstances regarding manufacturing scaleup, global distribution, and pathogen evolution can rapidly perturb successful vaccination deployments. Thus, the ongoing threat of ebolaviruses as well as the potential for future novel filovirus emergence events indicates the necessity to develop additional vaccine strategies for protection.

A major hurdle in the use of subunit vaccine platforms in development of an Ebola vaccine is the ability to produce large-scale quantities of Ebola GP as an antigen. GP, prior to cleavage of the mucin-like domain and the glycan cap, is heavily glycosylated and expressed recombinantly in mammalian cells at low levels. However, removal of the mucin-like domain has been shown to enhance expression levels considerably without affecting protein stability and folding (18), and pseudoviruses expressing the GPΔmucin form of GP are capable of host cell entry. Furthermore, antibody characterization studies have demonstrated that antibodies targeting the mucin-like domain are rarely capable of viral neutralization, suggesting that deletion of this domain should not affect elicitation of neutralizing antibodies in the context of vaccination. Thus, in our antigen designs we chose to eliminate the mucin-like domain to enhance protein expression levels and create antigens containing only those antigenic domains of GP known to be targets of neutralizing antibodies.

Fusion of GPΔmucin to both the ferritin and E2p subunit yielded functionalized nanoparticle constructs that expressed at equivalent levels to the GPΔmucin trimer (**Figure 1B**). Additionally, the GPΔmucin functionalized nanoparticles were highly homogenous following purification as determined *via* SEC-MALS (**Figure 1D**), indicating that they were not prone to either aggregation or particle dissociation. We used differential scanning fluorimetry to confirm fusion of GPΔmucin to a nanoparticle did not perturb thermodynamic stability and determined *via* this method that the melting temperature of GPΔmucin on ferritin and E2p nanoparticles was equivalent to that of GPΔmucin trimer (**Figure 2A**). Additionally, we confirmed homogeneity of the nanoparticle scaffold by performing cryo-EM and assessing the 2D class averages of GP-Fer and GP-E2p (**Figure 2B**). Finally, we confirmed that antigenicity of critical epitopes on GPΔmucin were not perturbed by display on multivalent scaffolds using BLI binding assays. As seen in **Figure 2C**, conformation specific antibodies bind all three GPΔmucin antigens with similar on-rates, suggesting that these epitopes are maintained even in the context of multimerization.

In comparing the *in vivo* immune responses to our panel of GPΔmucin antigens, we observed that antigen multimerization was important for the rate of antigen-specific antibody elicitation, but played a minimal role in neutralizing antibody levels. These results are consistent overall with a recent study that also investigated the effects of nanoparticle presentation of GPΔmucin on antibody responses following vaccination (15). Interestingly, the GPΔmucin antigen-specific titers observed at week 2 following a single dose in that study are all nearly equivalent, showing little benefit of nanoparticle multimerization (15). The titers we observe at this same timepoint (**Figure 3B**, week 2), shows a ~2-3 fold enhancement in antibody levels in both GP-Fer and GP-E2p groups compared to GP-GCN4. This discrepancy could be due to a difference in adjuvant composition, since we utilized Quil-A+MPLA and He et al. administered their antigens formulated with AddVax or Adju-Phos. Additionally, the dose used by He et al. was 50 µg of antigen per mouse, whereas we administered 5 µg antigen. Thus, it could be possible that effects of multimerization are less substantial at higher doses of antigen or with different combinations of adjuvants. Furthermore, a key caveat to the immunization data presented here is that multimerization was only tested in the context of one adjuvant combination (Quil-A+MPLA). It is possible that formulation with other adjuvants could demonstrate a differing role of multimerization on the elicited antibody response.

Importantly, our data clearly demonstrate that serum from mice immunized with GPΔmucin antigens in this study does not enhance infectivity of GP-pseudotyped lentiviruses. Recently published work (15) suggested that immunization of rabbits and mice with Ebola GP subunit vaccines could elicit antibodies capable of enhancing GP-pseudotyped lentiviral entry into mammalian cells. To confirm that this was not occurring in our study, we assessed serum neutralization levels to serum dilutions of ~10^7^ (**Supplementary Figure S3**). Analysis of serum from GP-GCN4, GP-Fer, and GP-E2p immunized mice revealed that even at the lowest concentrations, viral infectivity plateaued at ~100%, therefore suggesting that serum antibodies did not possess the capability of binding to GP-pseudotyped lentiviral particles and enhancing viral infectivity. Discrepancies between our findings and those recently reported (15) are likely due to technical variations in the pseudovirus neutralization assays performed with serum from immunizations. As reported in (15), the authors observe considerable enhancement of infectivity in their non-target control virus samples with both mouse and rabbit serum, suggesting non-specific interactions between the serum components and the pseudotyped viruses irrespective of the surface protein. Thus, it is unlikely that antibody dependent enhancement (ADE) will arise as an issue in the development of subunit-based vaccines against Ebola.

In assessing the temporal aspect of the antibody response post vaccination, we observe that antigen-specific binding titers are maximum at 3 weeks following a single dose for all three antigen groups (**Figure 3B**). Interestingly, the neutralizing antibodies do not appear to decline as antigen-specific titers do, (compare **Figures 3B**, **4B**). Between weeks 6 and 18, antigen-specific ELISA titers decrease by 2-3 fold for all three antigen groups whereas the percent neutralization for both GPΔmucin and FL GP pseudotyped viruses at 1:300 appear to be the same at these time points. This could suggest a durable and persistent neutralizing antibody response against Ebola can be elicited with a single adjuvanted subunit vaccine dose and will be an interesting point to assess in future studies.

A common approach for subunit vaccine design is to use a domain or fragment of an antigen known to be the target of neutralizing antibodies and use that as an immunogen. While this is successful in some cases, there can be limitations to this approach. For example, the SARS-CoV-2 spike receptor binding domain is an immunodominant epitope known to be the target of highly potent neutralizing antibodies following both infection and vaccination (42–44). However, in previous studies, we and others (31, 32) have demonstrated that a single dose of receptor binding domain compared to full-length spike and spike nanoparticles elicited significantly weaker overall neutralizing antibody responses (31, 32).

For Ebola subunit vaccines, previous work suggested that GPΔmucin antigens could elicit neutralizing antibody levels equivalent to FL GP antigens (45). Despite this evidence and the finding that the mucin-like domain is rarely the target of neutralizing antibodies (25), we observed that both antigen-specific titers and neutralizing antibody titers against FL GP were much lower than those elicited to GPΔmucin. This suggests that using GPΔmucin as an antigen favored elicitation of antibodies that can bind to epitopes exposed in the context of GPΔmucin, but are unable to bind to FL GP. Therefore, while the mucin-like domain antibodies have been characterized primarily as non-neutralizing, the presence of this domain could still be essential for eliciting neutralizing antibodies that target epitopes on adjacent domains. Its removal may lead to elicitation of antibodies to non-biologically relevant epitopes that are not exposed on the native pathogen. This is an important consideration in designing future iterations of Ebola subunit vaccines and may require design of a more stable FL GP trimer that can be expressed at higher levels.

Design and development of protein nanoparticle vaccines are an exciting new strategy in the field of vaccinology and are showing great promise in pre-clinical studies and early clinical trials. For many viruses, multimerization of an antigen leads to dramatically enhanced neutralizing antibody responses following vaccination. Interestingly, the results we present here as well as those presented in recently published work (15), suggest that multimerization does not significantly boost antibody responses in the context of GPΔmucin. It is possible that multimerization would enhance neutralizing antibody responses with GPΔmucin after a second dose, which was not tested here. Our results could suggest that the overall mechanisms of antibody-mediated neutralization vary from those of respiratory viruses such as SARS-CoV-2, RSV, and influenza compared to ebola viruses. For example, it is possible that multimerized antigens may elicit antibodies more prone to bridge multiple surface antigens, which could result in enhanced neutralization of some viruses and not others. Given that Ebola viruses sheds the mucin-like domain the MLD on the surface glycoprotein, this may suggest that trimer-bridging antibodies are less relevant to neutralization of this virus. Alternatively, in our specific study, the lack of effect of multimerization could be due to the use of GPΔmucin instead of the FL GP with the mucin-like domain intact. Future nanoparticle designs using FL GP could help to elucidate this.

## Data availability statement

The raw data supporting the conclusions of this article will be made available by the authors, without undue reservation.

## Ethics Statement

The animal study was reviewed and approved by Stanford University Administrative Panel on Laboratory Animal Care.

## Author contributions

All authors contributed to experimental design. AP and GR performed mouse work. AEP performed antigen purification and characterization, serum ELISAs, and data analysis. AP and DX performed pseudovirus neutralization assays. KZ performed cryo-EM sample preparation, data collection, and image processing. AP and PK wrote the manuscript. All authors assisted with editing and approved the final version of the manuscript.

## Funding

This work was supported by the Stanford Maternal and Child Health Research Institute postdoctoral fellowship (to AP), the National Institutes of Health (DP1AI158125), the Chan Zuckerberg Biohub (to WC and PK), and the Virginia and D. K. Ludwig Fund for Cancer Research (to PK).

## Acknowledgments

We thank members of the Kim Lab for fruitful discussions and insight on project design as well as helpful comments on the manuscript. We thank Dr. Jesse Bloom and Kate Crawford for providing the lentiviral plasmids for pseudotyped virus production. The CMV/R antibody expression vectors were received from the NIH AIDS Reagent Program.

## Conflict of Interest

The authors declare that the research was conducted in the absence of any commercial or financial relationships that could be construed as a potential conflict of interest.

## Publisher’s note

All claims expressed in this article are solely those of the authors and do not necessarily represent those of their affiliated organizations, or those of the publisher, the editors and the reviewers. Any product that may be evaluated in this article, or claim that may be made by its manufacturer, is not guaranteed or endorsed by the publisher.
