# Supplementary material for: Multimerization of Ebola GPΔmucin on protein nanoparticle vaccines has minimal effect on elicitation of neutralizing antibodies
Source: Front Immunol. 2022 Aug 24;13:942897. doi: 10.3389/fimmu.2022.942897 (PMC9449635; doi:10.3389/fimmu.2022.942897)
Supplement: Supplementary file 1 [file Presentation_1.pptx]

## Slide 1
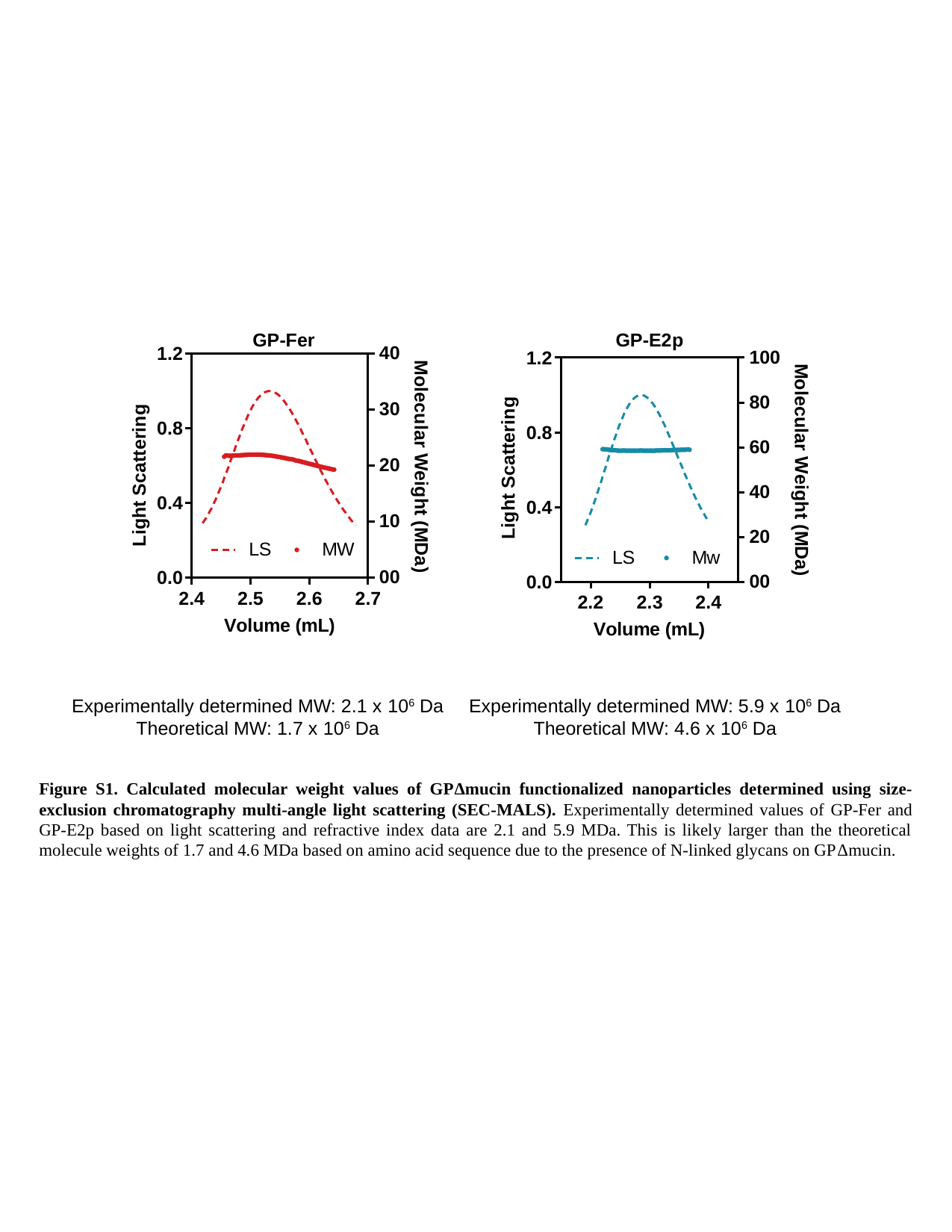

Experimentally determined MW: 2.1 x 10^6^ Da
Theoretical MW: 1.7 x 10^6^ Da
Experimentally determined MW: 5.9 x 10^6^ Da
Theoretical MW: 4.6 x 10^6^ Da
Figure S1. Calculated molecular weight values of GPΔmucin functionalized nanoparticles determined using size-exclusion chromatography multi-angle light scattering (SEC-MALS). Experimentally determined values of GP-Fer and GP-E2p based on light scattering and refractive index data are 2.1 and 5.9 MDa. This is likely larger than the theoretical molecule weights of 1.7 and 4.6 MDa based on amino acid sequence due to the presence of N-linked glycans on GPΔmucin.

## Slide 2
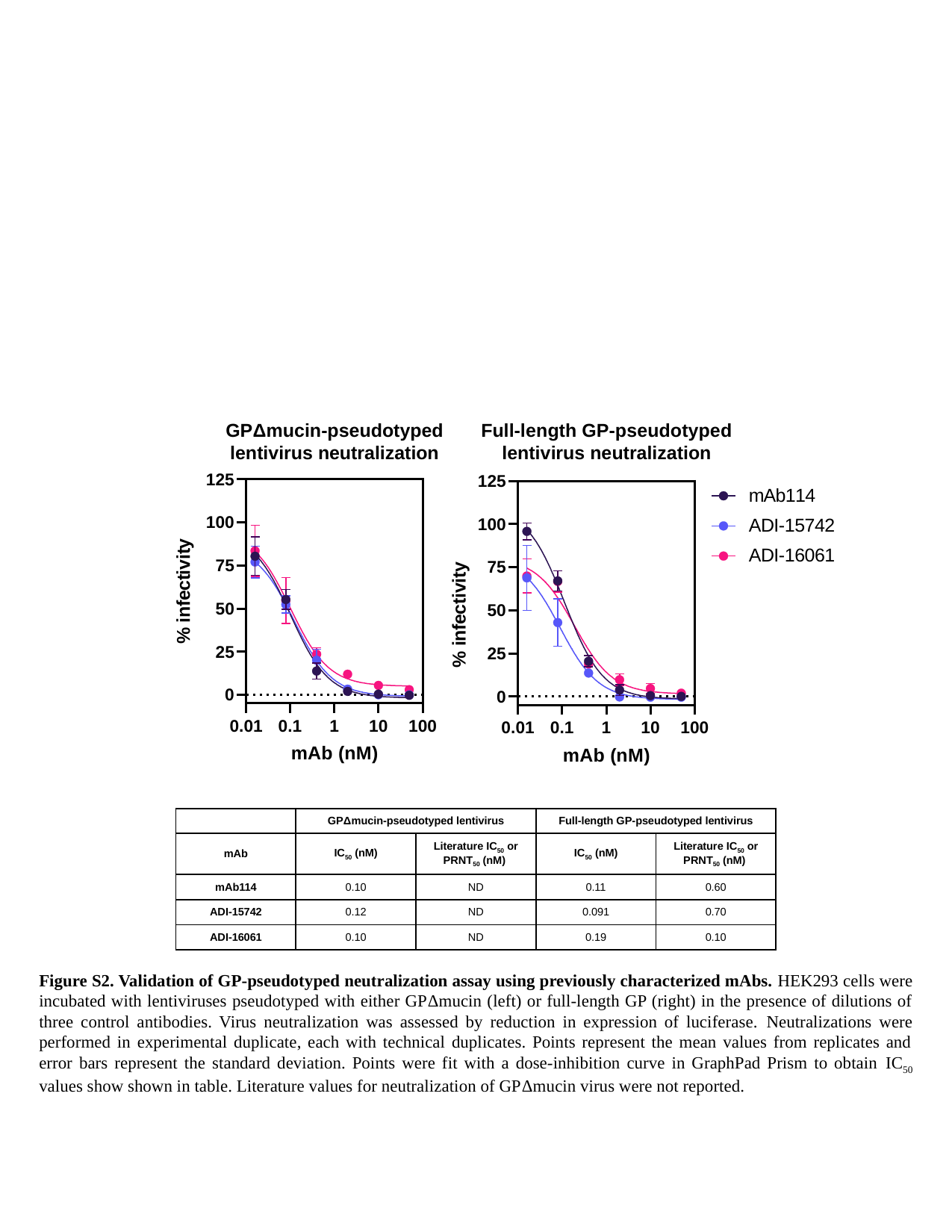

| | GPΔmucin-pseudotyped lentivirus | | Full-length GP-pseudotyped lentivirus | |
| --- | --- | --- | --- | --- |
| mAb | IC50 (nM) | Literature IC50 or PRNT50 (nM) | IC50 (nM) | Literature IC50 or PRNT50 (nM) |
| mAb114 | 0.10 | ND | 0.11 | 0.60 |
| ADI-15742 | 0.12 | ND | 0.091 | 0.70 |
| ADI-16061 | 0.10 | ND | 0.19 | 0.10 |
Figure S2. Validation of GP-pseudotyped neutralization assay using previously characterized mAbs. HEK293 cells were incubated with lentiviruses pseudotyped with either GPΔmucin (left) or full-length GP (right) in the presence of dilutions of three control antibodies. Virus neutralization was assessed by reduction in expression of luciferase. Neutralizations were performed in experimental duplicate, each with technical duplicates. Points represent the mean values from replicates and error bars represent the standard deviation. Points were fit with a dose-inhibition curve in GraphPad Prism to obtain IC50 values show shown in table. Literature values for neutralization of GPΔmucin virus were not reported.

## Slide 3
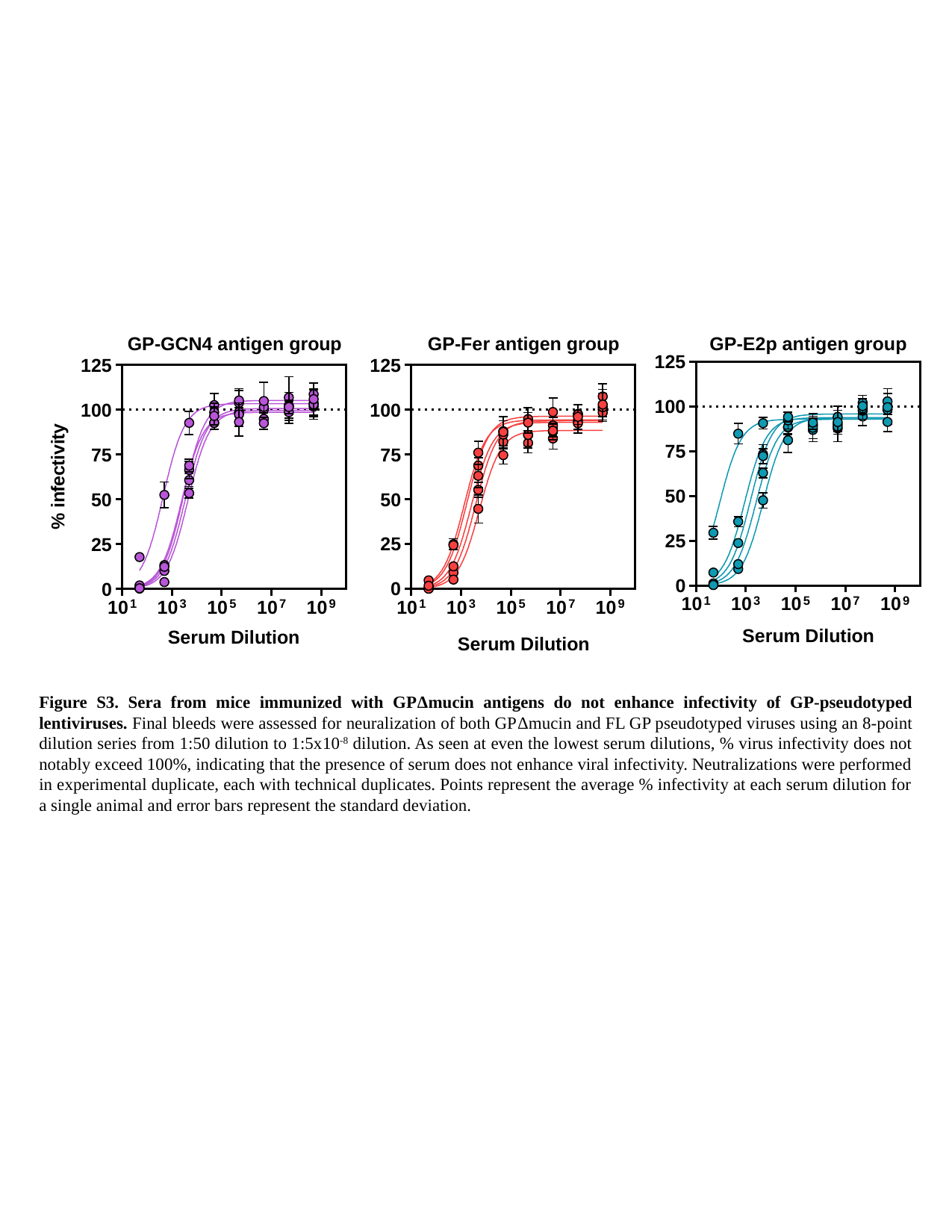

Figure S3. Sera from mice immunized with GPΔmucin antigens do not enhance infectivity of GP-pseudotyped lentiviruses. Final bleeds were assessed for neuralization of both GPΔmucin and FL GP pseudotyped viruses using an 8-point dilution series from 1:50 dilution to 1:5x10^-8^ dilution. As seen at even the lowest serum dilutions, % virus infectivity does not notably exceed 100%, indicating that the presence of serum does not enhance viral infectivity. Neutralizations were performed in experimental duplicate, each with technical duplicates. Points represent the average % infectivity at each serum dilution for a single animal and error bars represent the standard deviation.
